# Clinical Characteristics Associated with *Borrelia burgdorferi* Sensu Lato Skin Culture Results in Patients with Erythema Migrans

**DOI:** 10.1371/journal.pone.0082132

**Published:** 2013-12-26

**Authors:** Franc Strle, Lara Lusa, Eva Ružić-Sabljić, Vera Maraspin, Stanka Lotrič Furlan, Jože Cimperman, Katarina Ogrinc, Tereza Rojko, Jerneja Videčnik Zorman, Daša Stupica

**Affiliations:** 1 Department of Infectious Diseases, University Medical Center Ljubljana, Ljubljana, Slovenia; 2 Institute for Biostatistics and Medical Informatics, Faculty of Medicine Ljubljana, Ljubljana, Slovenia; 3 Institute of Microbiology and Immunology, Faculty of Medicine Ljubljana, Ljubljana, Slovenia; University of Kentucky College of Medicine, United States of America

## Abstract

Clinical characteristics associated with isolation of *Borrelia burgdorferi* sensu lato from skin have not been fully evaluated. To gain insight into predictors for a positive EM skin culture, we compared basic demographic, epidemiologic, and clinical data in 608 culture-proven and 501 culture-negative adult patients with solitary EM. A positive *Borrelia* spp. skin culture was associated with older age, a time interval of >2 days between tick bite and onset of the skin lesion, EM ≥5 cm in diameter, and location of the lesion on the extremities, whereas several other characteristics used as clinical case definition criteria for the diagnosis of EM (such as tick bite at the site of later EM, information on expansion of the skin lesion, central clearing) were not. A patient with a 15-cm EM lesion had almost 3-fold greater odds for a positive skin culture than patients with a 5-cm lesion. Patients with a free time interval between the tick bite and onset of EM had the same probability of a positive skin culture as those who did not recall a tick bite (OR=1.02); however, the two groups had >3-fold greater odds for EM positivity than patients who reported a tick bite with no interval between the bite and onset of the lesion. In conclusion, several yet not all clinical characteristics used in EM case definitions were associated with positive *Borrelia* spp. skin culture. The findings are limited to European patients with solitary EM caused predominantly by *B. afzelii* but may not be valid for other situations.

## Introduction

The isolation and identification of a microorganism is the gold standard for demonstration of the etiology of an infectious disease. This general rule applies also to Lyme borreliosis (LB), where the cultivation of borreliae from clinical specimens is a time-consuming procedure with limited sensitivity. The isolation rate of *Borrelia* spp. from human specimens depends on the laboratory approaches to cultivation and isolation, and on several clinical factors such as the accuracy of LB diagnosis, the clinical manifestation of the disease, and the source, quality, and quantity of specimens obtained for cultivation. The isolation rate is highest for skin specimens of erythema migrans (EM) lesions. In series including >100 patients with EM, published during the past 15 years, the isolation rate has been 43–60% [1–7]. 

The large numbers of patients with EM seen at our LB outpatient clinic and the high proportion of patients who consent to skin biopsy have enabled insight into associations between clinical characteristics and positive skin culture results.

## Patients and Methods

### Ethic statement

The approach to the management of patients that enabled collection of data for the present report was approved by the Medical Ethics Committee of the Ministry of Health of the Republic of Slovenia (No 38/05/06). The investigation was conducted according to the principles expressed in the Declaration of Helsinki. Signed written informed consent was obtained from all study participants. 

### Patients

All patients >15 years old, examined for EM at the LB outpatient clinic of the Department of Infectious Diseases, University Medical Center Ljubljana, Slovenia, between 2005 and 2008, were evaluated for participation in the study. The large majority were referred from general physicians. Patients who had other obvious clinical explanations for their skin lesions, such as reaction to insect bites, fungal skin infections, contact eczema, folliculitis, cellulitis, or fixed drug eruption, were not eligible for participation. Patients were diagnosed with EM according to the Slovenian definition (Table 1) [10]. Several patients with lesions ≥5 cm who did not recall expansion (usually because they noticed the lesion only recently) but who fulfilled all the other criteria were also included in the analysis.

**Table 1:** Main characteristics required for the diagnosis of erythema migrans skin lesions according to differing case definitions.

**Requirements**	**Definitions**				
	CDC [8]	Stanek et al. [9]	Strle et al. [10]	Brouqui et al. [11]	Stanek et al. [12]
Expansion	+	+	+	+	+
Central clearing	+ (often)	+ (often)	+ (often)	+ (often)	–
Diameter ≥5 cm	+	+ (most cases)	+	-	+
Additional systemic symptoms	+ (most patients)	+ (may be present)	–	–	–
Tick bite	–	+ (risk of exposure to ticks)	+^a^	+^b^	+^c^
Delay in appearance^c^	–	–	+^a^	–	+^a^
Delay in appearance^c^ >2 days	–	–	–	–	+^a^

+, parameter required; –, parameter not required.

^a^ Criterion required only for lesions with the largest diameter <5 cm.

^b^ Minor criterion.

^c^ Delay in appearance after a tick bite.

At the initial visit, the history was taken and clinical examination performed. Demographic, epidemiologic, and clinical data were obtained using a structured questionnaire. Precise descriptions of the evolution of the EM, the presence of accompanying symptoms, information on previous antibiotic treatment, tick bites, and LB in the past were of special interest. In clinical examination, particular attention was paid to the skin lesion and other signs potentially associated with LB. 

Patients were excluded if they had any other objective manifestations of LB in addition to EM (such as involvement of nervous system or joints) and/or had >1 EM lesion, it they did not consent to skin biopsy, if they had had LB previously and/or received an antibiotic with known anti-borrelial activity within 10 days prior to examination.

Data on a subset of 252 patients diagnosed with EM in 2006 (including 151 patients with isolation of borreliae from skin) have been reported for distinct purposes elsewhere [4,13]. 

### Skin culture of borreliae

The skin specimen (2.5 x 2 x 2 mm) obtained from the expanding edge of the EM was placed in 10 ml of modified Kelly-Pettenkofer culture medium as described previously [14]. Cultures were examined weekly for the presence of spirochetes by dark-field microscopy and were interpreted as negative if no growth was established after 9 weeks. 

### Statistical analysis

To gain insight into potential predictors for a positive EM skin culture, we assessed differences in basic demographic, epidemiologic, and clinical data between culture-positive and culture-negative groups of patients. 

Data were summarized as medians with interquartile range (IQR) for numerical variables and as frequencies and percentages for categorical variables. Logistic regression models were used to assess the association between each potential predictor and EM culture positivity, adjusting the analysis for the size of EM. Restricted cubic splines [15] were used to flexibly model the relationship between culture positivity and largest diameter of EM and seasonal occurrence, thus avoiding the a priori assumption of linearity (on the logit scale) between the continuous variables and culture positivity. The other numerical variables were modeled linearly. Results were presented as unadjusted and adjusted odds ratios (ORs) and *P* values. 

The combined effect of the characteristics required for the diagnosis of EM (according to different case definitions) on culture positivity was evaluated using a multivariable logistic regression model.

The shape of the association between the probability of a positive *Borrelia* spp. skin culture and several numerical variables (patient age, time lapse between tick bite and biopsy, duration of the lesion prior to biopsy, the largest diameter of EM, calendar date of skin biopsy) was estimated using restricted cubic splines and univariate logistic regression; results are represented graphically. 

Univariate linear regression models were also used to assess the association of size of EM with age and seasonal occurrence, using restricted cubic splines; similar models were fitted using duration of EM as outcome.

R statistical language was used for the statistical analyses [16].

## Results

In the 4-year period 2005–2008, EM without any clinically evident extracutaneous signs of LB was diagnosed in a total of 2042 patients >15 years old. 

**Figure 1:**
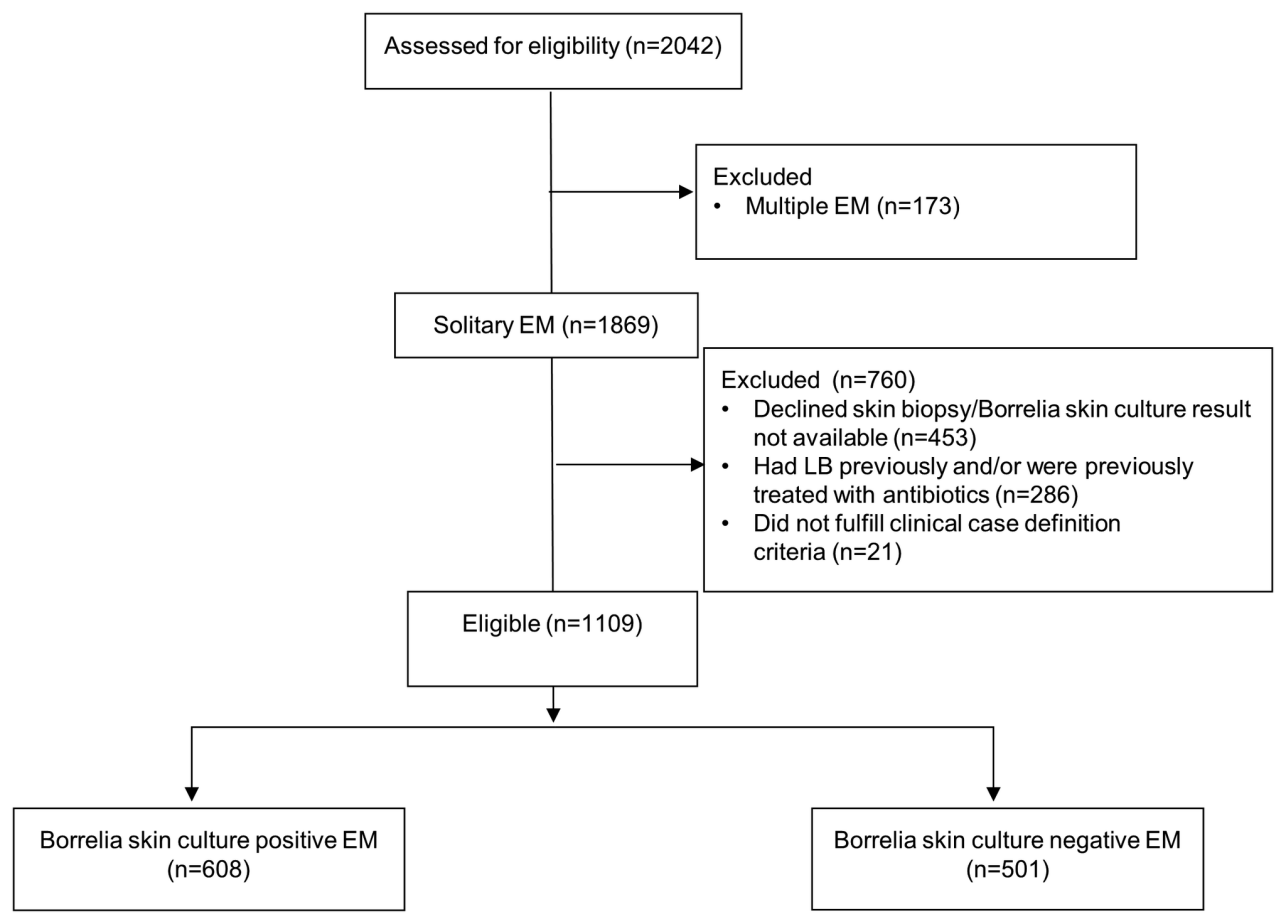


**Figure 2:**
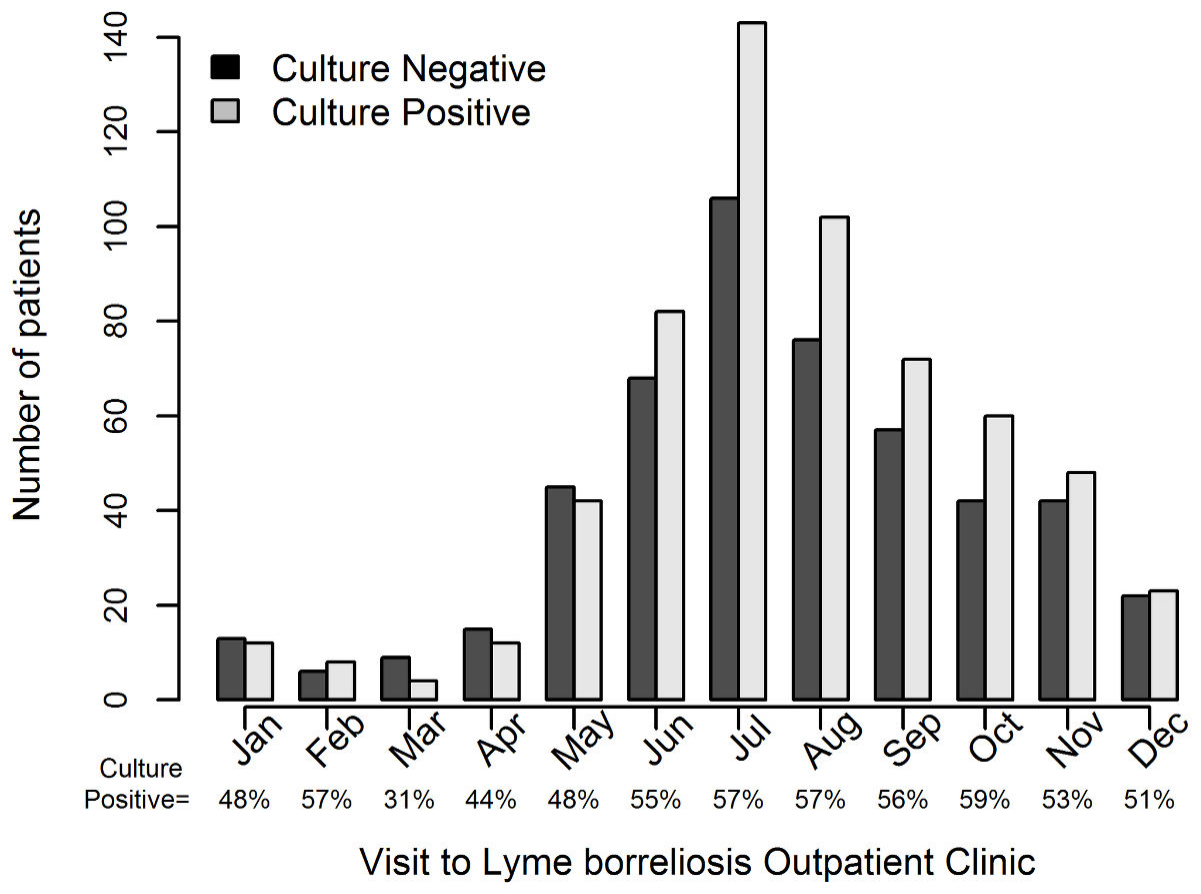


In this analysis we focused on 1109 patients (608 culture positive [55%], 501 culture negative) who fulfilled the inclusion criteria and had no exclusion criteria (Figure 1). The large majority of these patients were diagnosed with EM between June and October (Figure 2).

Comparison of culture-positive and culture-negative patients (Table 2) showed that those with a positive *Borrelia* spp. skin culture were older (the isolation rate increased with rising age: the odds of positive culture increased about 10% for a 10-year age increase), had more frequently an interval of >2 days between the tick bite and the onset of EM, had more often an EM ≥5 cm in diameter, had a larger diameter of the skin lesion, and had EM more often localized on the extremities than on other body sites. 

**Table 2:** Characteristics of patients with culture-positive and culture-negative erythema migrans.

**Characteristic**	**Culture positive**	**Culture negative**	Unadjusted*		Adjusted for size of EM**	
	**(n=608)**	**(n=501)**	**OR**	***P***	**OR**	***P***
Age (years)	52 (40–60)	50 (36–59)	**1.10** ^α^	0.03	**1.10** ^α^	0.03
Male sex	275 (45.2%)	221 (44.1%)	1.05	0.71	0.99	0.94
Tick bite	333 (54.6%)	287 (57.3%)	0.90	0.40	0.97	0.85
Days from tick bite to onset of EM^a^	17 (10–29)	14 (7–27)	1.00	0.51	1.00	0.68
>2 days from tick bite to onset of EM^a^	296 (94.6%)	235 (86.4%)	**2.74**	<0.001	**2.70**	<0.001
Duration of EM (days)^b^	9 (4–30)	10 (4–23)	**0.95** ^β^	<0.001	**0.94** ^β^	<0.001
Central clearing of EM^c^	346 (57.0%)	272 (54.3%)	1.12	0.37	0.98	0.86
Largest diameter of EM (cm)^d,γ^	14 (10–21)	12 (8–23)		<0.001	--	--
10 vs. 5 cm			**1.80**			
15 vs. 5 cm			**2.95**			
20 vs. 5 cm			**2.41**			
30 vs. 5 cm			**1.60**			
40 vs. 5 cm			**1.38**			
Largest diameter of EM ≥5 cm^d^	597 (98.8%)	484 (96.6%)	**3.00**	0.02	--	--
Expansion of skin lesion (reported by patients)^e^	366 (63.0%)	287 (58.6%)	1.20	0.14	1.12	0.40
Ratio of EM diameters^f^	1.53 (1.22–2)	1.46 (1.19–1.89)	1.06	0.38	1.04	0.54
EM on leg or arm	509 (84.0%)	382 (76.2%)	**1.64**	0.001	**1.59**	0.003
Presence of non-specific symptoms^c,g^	176 (29.0%)	147 (29.3%)	0.98	0.90	1.00	0.99
Presence of symptoms at EM site^g^	287 (47.2%)	246 (49.1%)	0.93	0.53	0.93	0.55
Seasonal occurrence^h,γ^				0.28		0.41
July vs. April			1.25		1.25	
July vs. October			0.98		0.97	
July vs. January			1.47		1.42	

Data are median (interquartile range) or number (%); EM, erythema migrans; OR, odds ratio. OR and *P* values were obtained using *univariate logistic regression or **multiple logistic regression, adjusting the analysis for the size of EM (modeled using restricted cubic splines).

^a^ Patients with history of a tick bite at the site of the EM skin lesion. The information is missing for 20 culture-positive and 15 culture-negative patients.

^b^ Information missing for 1 culture-positive patient and 2 culture-negative patients.

^c^ Information missing for 1 culture-positive patient.

^d^ Information missing for 4 culture-positive patients.

^e^ Information missing for 27 culture-positive and 11 culture-negative patients.

^f^ Information missing for 2 culture-positive patients.

^g^ Some patients had more than one symptom.

^h^ Seasonal occurrence of skin biopsy was defined as the number of days between the visit to the Lyme borreliosis outpatient clinic and January 1^st^ of the same year.

^α^ OR for 10-year increase in age.

^β^ OR for 7-day increase in EM duration.

^γ^ Restricted cubic splines were used to flexibly model the relationship between the covariate and culture positivity. Estimated ORs comparing different values are reported for descriptive purposes only.

No differences were established for several other parameters, including some that are criteria for the diagnosis of EM, such as reporting a tick bite, expansion of the skin lesion, central clearing, and the presence of systemic symptoms. After adjustment of the analysis for the size of EM, the findings of the comparisons remained similar (Table 2). 

When the characteristics required for a diagnosis of EM according to case definitions were analyzed together (Table 3), EM ≥5 cm in diameter remained significantly associated with culture positivity (OR=2.9, *P*=0.02). Patients with a free time interval between the tick bite and onset of EM had the same probability of a positive skin culture as those who did not recall a tick bite (OR=1.02); however, both groups had >3-fold greater odds for EM positivity than patients who reported a tick bite with no interval between the bite and onset of the lesion. Expansion of EM was associated with a 20% increase in the odds for EM culture positivity but the association was nonsignificant (*P*=0.14).

**Table 3:** Association between the main characteristics required for the diagnosis of erythema migrans skin lesion and culture positivity, estimated using multiple logistic regression.

**Requirements**	**OR^a^ (95% CI)**	***P***
Expansion	1.21 ( 0.94; 1.55)	0.14
Central clearing	1.04 ( 0.82; 1.34)	0.73
Largest diameter of erythema migrans ≥5 cm	2.93 (1.19; 7.20)	0.02
Presence of non-specific symptoms	0.96 (0.74; 1.25)	0.77
Tick bite		0.07
Bite with delay in appearance vs. no bite	1.02 (0.79; 1.31)	
Bite without delay in appearance vs. no bite	0.29 (0.09; 0.92)	

^a^ Each odds ratio (OR) is adjusted for all the other variables in the table.

**Figure 3:**
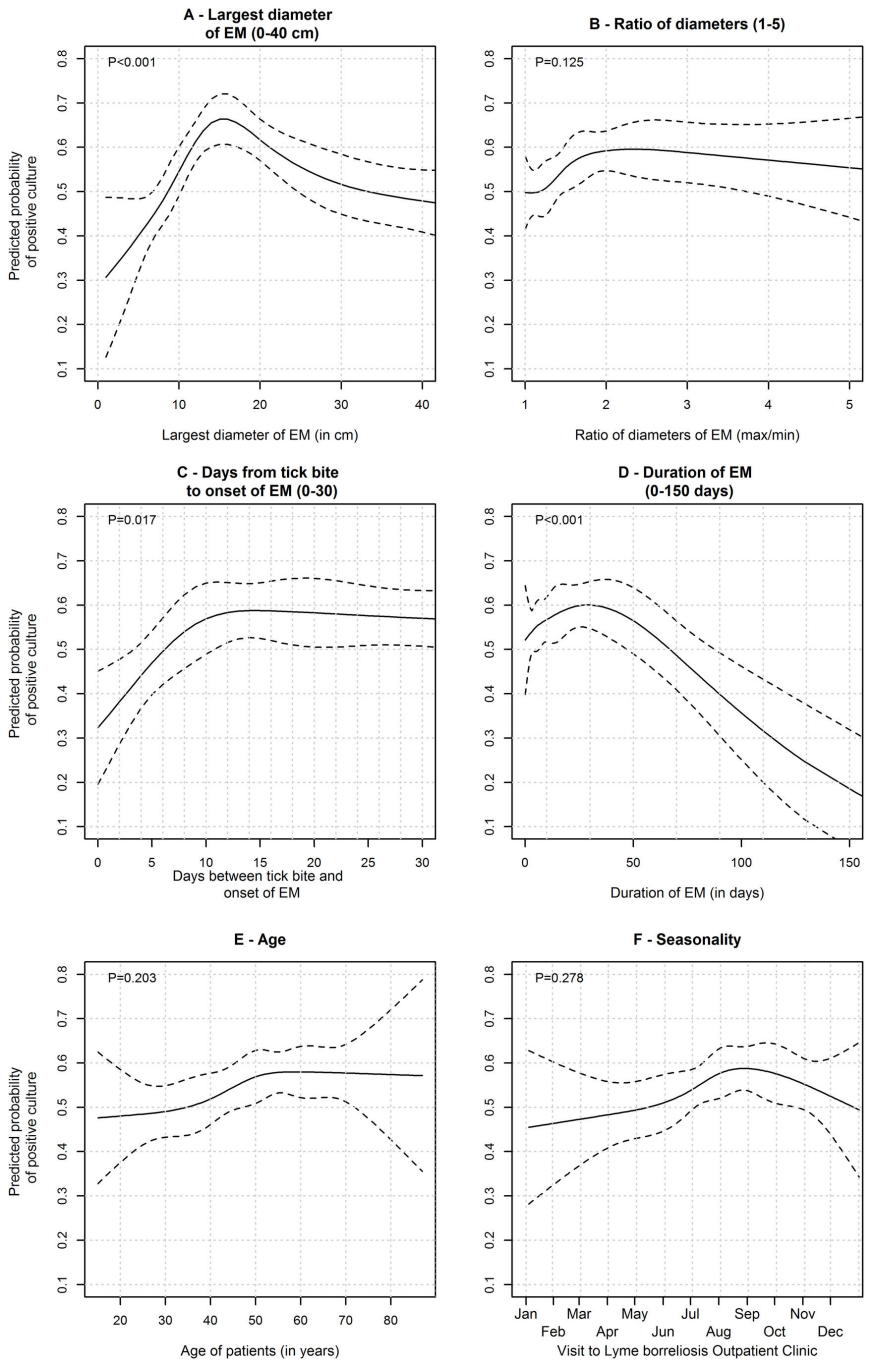


**Figure 4:**
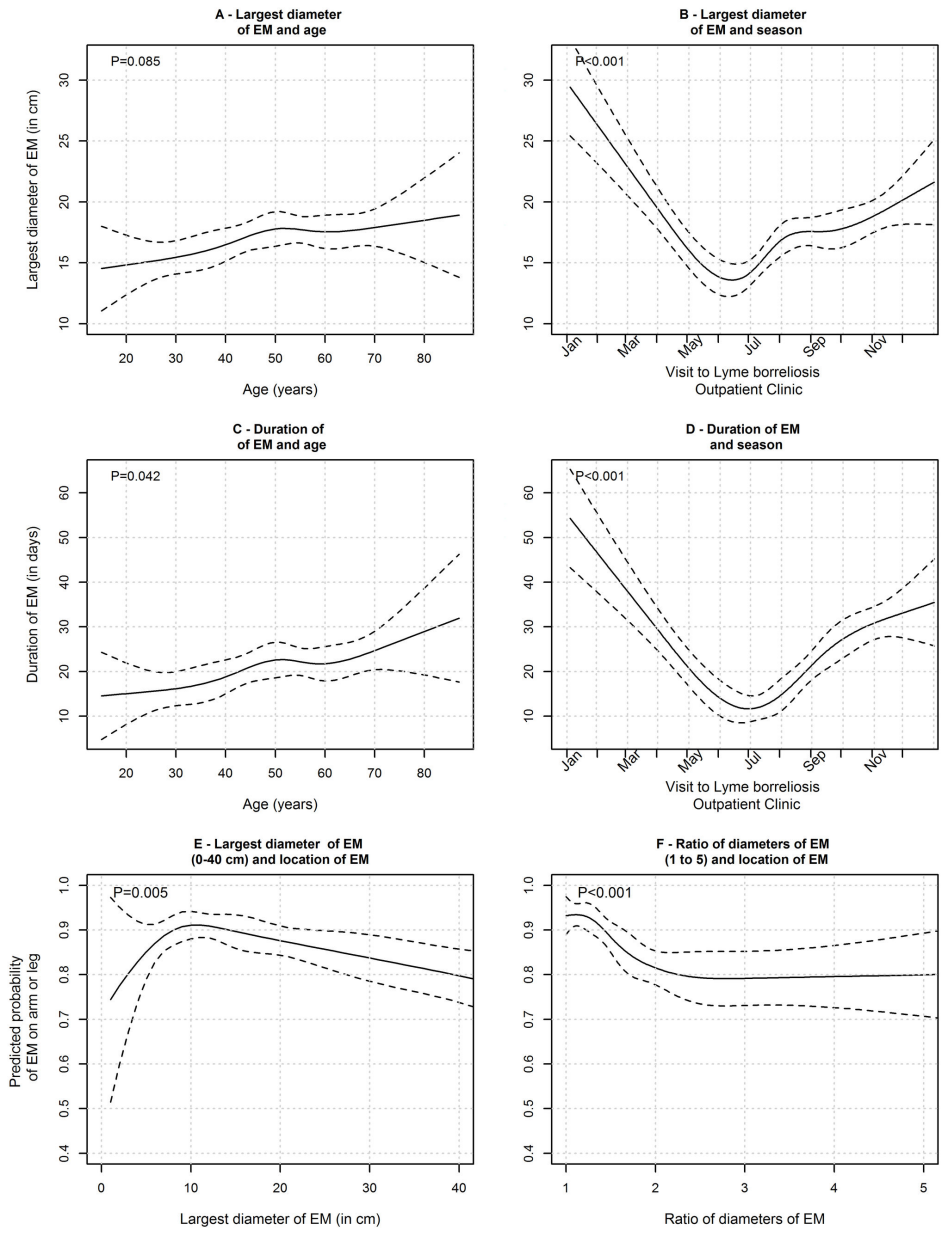


We determined the shape of the association between various numerical variables and the probability of a positive culture result. As illustrated in Figure 3, the probability of a positive culture markedly increased with the rising size of the largest diameter of EM up to about 15 cm, decreasing slowly afterwards. The estimated probability of a positive culture was lowest for circular lesions and highest for elliptical lesions with diameter ratios 2:1 to 3:1, although the difference was not significant (Table 2, Figure 4). The probability showed a slight increase for lesions with duration up to 30 days, pronouncedly decreasing for long-lasting EMs. Borreliae were more often isolated from the skin of older patients than younger ones; there was no evidence of a non-monotonic association between age and probability of culture positivity (*P*=0.20, Figure 3). The probability of having a positive culture result was highest between June and October and later diminished until the next tick season (Figures 2 and 3), but the association between seasonality and culture positivity was not statistically significant (*P*=0.28). 

To gain greater insight into some of these rather unexpected findings we also assessed characteristics of EM according to age of patients, seasonal appearance, and location of the lesion (Figure 4). The duration of EM prior to diagnosis increased with rising age, as did the size of the skin lesion. The proportion of patients with small and relatively short-lasting EM was highest at the end of spring and in early summer (the beginning of the tick season in Slovenia), whereas patients with large, long-lasting lesions were diagnosed during late autumn and winter (Figure 4). 

## Discussion

Diagnosis of EM is clinical. According to guidelines no microbiological verification is needed for the diagnosis [17,18]. Nonetheless, patients who fulfill the clinical criteria and have borreliae isolated from the skin lesion represent the group with the most substantiated diagnosis. If a clinical definition lacks specificity, patients with falsely diagnosed EM will accumulate in the culture-negative group and this will most probably result in distinctions between patients with positive and negative skin cultures in at least some of the characteristics. However, in addition to misdiagnosing, several other factors may contribute to negative culture results. 

As shown in Table 1, clinical definitions of EM require several criteria to be met, the most consistent being expansion of the lesion. Although this criterion appears reasonable because it follows the natural course of EM, it cannot be always fulfilled in routine clinical practice. In the present study, 39% of patients presented with rather large skin lesions that they had noticed only recently. Thus, such lesions “did not have enough time” to enlarge noticeably and could not be formally appreciated as increasing. The problem may be more pronounced in Europe, where the majority of EM is due to *Borrelia afzelii*, a strain causing slow-growing EM lesions that are relatively infrequently accompanied by systemic symptoms, unlike the situation in North America, where the only etiologic agent is *B. burgdorferi* sensu stricto – this lesion enlarges faster and is more often associated with pronounced systemic symptoms than EM caused by *B. afzelii* [19]. In the present study the numbers of patients reporting expansion of the lesion were similar in the culture-positive and culture-negative groups (63.5% vs. 58.6%, *P*=0.40), indicating that this parameter had no substantial influence on the accuracy of diagnosis in our patients.

Some clinical definitions of EM require that the lesion has a diameter ≥5 cm [8–10,12], other definitions do not [11]. In the present study the odds of isolating borreliae from skin were 3 times higher for lesions ≥5 cm than for smaller ones. However, the highest rate of isolation from the expanding border of EM was established for lesions with largest diameter of about 15 cm (Figure 3). A patient with a 15-cm lesion had almost 3-fold greater odds for a positive skin culture than those with a 5-cm EM, i.e. the diameter used as a diagnostic criterion (Table 1). This finding is probably due to different concentration of borreliae according to EM diameter. Quantifying borrelia burden in EM with PCR revealed that the concentration is the highest in EM lesions with a diameter of 15-20 cm and that the isolation rate goes in parallel with the burden [7].

According to our results, an interval of >2 days between tick bite and onset of EM was another criterion that substantially contributed to skin culture positivity. When redness develops at the site of the attached tick (which happens in up to 1/3 persons with tick bite), the lesion most probably represents a local hypersensitivity reaction [8]. Such redness is usually <5 cm in diameter and disappears within a few days. However, in some patients the lesion does not vanish but enlarges for several days to reach a diameter well over 5 cm. Such a condition could be the result of a pronounced and prolonged local hypersensitivity reaction but could also be explained as an initial nonspecific hypersensitivity reaction followed unwaveringly by EM and resulting in the absence of an interval between tick bite and onset of EM. Although – according to our findings – such patients had >3 times lower odds for culture positivity than those with an interval of >2 days (Table 3), 31% (95% CI 20%–46%) of these patients were culture positive, suggesting that both options occur. 

It is a reasonable assumption that more patients are diagnosed with EM during warm months than in cold months but it was unexpected that the *Borrelia* spp. isolation rate was highest during the high season for EM (Figure 2). The latter finding is associated with variable characteristics of EM during the tick season. Thus, early in the season the large majority of patients have small, short-lasting skin lesions; in contrast, in late fall, winter, and early spring patients with long-lasting and large EM, infected several weeks to months earlier, are more frequent (Figure 4). According to our results (Figure 3), both small and very large lesions are associated with a low isolation rate of borreliae. 

We do not have a reliable explanation for the finding that *Borrelia* *spp.* culture-positive patients were older and had EM more often on extremities than on other body sites but would like to emphasize that these associations were established also after adjusting the analysis for the size of EM (Table 2). 

The characteristics of EM are influenced by the *Borrelia* species causing the lesion [19–21]. In the present study, we identified the species for a subset of 151 skin culture-positive patients [5] and found that 87% of the strains were *B. afzelii*, 8% *B. garinii*, and 5% *B. burgdorferi* sensu stricto. These ratios are congruent with an earlier report [14]. Our findings are therefore representative for (central) Europe but probably not for North America where the only *Borrelia* species causing LB is *B. burgdorferi* sensu stricto.

We emphasize that the findings presented here are limited to patients with solitary EM and may not be pertinent for the subgroups of patients with solitary EM that were excluded from our study or for patients with multiple EM and/or extracutaneous manifestations of LB.

In conclusion, our study in patients with EM has revealed that a positive *Borrelia* spp. skin culture was associated with older age, location of the lesion on the extremities, an interval of >2 days between tick bite and onset of the lesion, and EM ≥5 cm in diameter, but not with several other factors used as a diagnostic criteria in the majority of clinical case definitions for EM such as tick bite at the site of later EM, expansion of the skin lesion, and central clearing. 

## References

[1] Logar M, Lotric-Furlan S, Maraspin V, Cimperman J, Jurca T et al. (1999) Has the presence or absence of *Borrelia* *burgdorferi* sensu lato as detected by skin culture any influence on the course of erythema migrans? Wien Klin Wochenschr 111: 945–950. PubMed: 10666806. 10666806

[2] Ciceroni L, Ciarrochi S, Ciervo A, Mondarini V, Guzzo F et al. (2001) Isolation and characterization of *Borrelia* *burgdorferi* sensu lato strains in an area of Italy where Lyme borreliosis is endemic. J Clin Microbiol 39: 2254–2260. doi:10.1128/JCM.39.6.2254-2260.2001. PubMed: 11376066. 11376066 PMC88120

[3] Zore A, Ruzić-Sabljić E, Maraspin V, Cimperman J, Lotric-Furlan S et al. (2002) Sensitivity of culture and polymerase chain reaction for the etiologic diagnosis of erythema migrans. Wien Klin Wochenschr 114: 606–609. PubMed: 12422609. 12422609

[4] Cerar T, Ruzić-Sabljić E, Glinsek U, Zore A, Strle F (2008) Comparison of PCR methods and culture for the detection of *Borrelia* spp. in patients with erythema migrans. Clin Microbiol Infect 14: 653–658. doi:10.1111/j.1469-0691.2008.02013.x. PubMed: 18558937. 18558937

[5] Stupica D, Lusa L, Cerar T, Ružić-Sabljić E, Strle F (2011) Comparison of post-Lyme borreliosis symptoms in erythema migrans patients with positive and negative *Borrelia* *burgdorferi* sensu lato skin culture. Vector Borne Zoonotic Dis 11: 883–889. doi:10.1089/vbz.2010.0018. PubMed: 21083376. 21083376

[6] Stupica D, Lusa L, Cerar T, Ružić-Sabljić E, Strle F (2012) Treatment of erythema migrans with doxycycline for 10 days versus 15 days. Clin Infect Dis 55: 343 –350. doi:10.1093/cid/cis402. PubMed: 22523260. 22523260

[7] O’Rourke M, Traweger A, Lusa L, Stupica D, Maraspin V, et al. (2013) Quantitative detection of *Borrelia* *burgdorferi* sensu lato in erythema migrans skin lesions using internally controlled duplex real time PCR. PLOS One 8(5): e63968. doi:10.1371/journal.pone.0063968. Accessed 30 August 2013 23696863 PMC3655952

[8] CDC (1997) Case definitions for infectious conditions under public health surveillance. MMWR 46 (No. RR–10) 9148133

[9] Stanek G, O'Connell S, Cimmino M, Aberer E, Kristoferitsch W et al. (1996) European Union Concerted Action on risk assessment in Lyme borreliosis: Clinical case definitions for Lyme borreliosis. Wien Klin Wochenschr 108: 741–747. PubMed: 8990511. 8990511

[10] Strle F, Videcnik J, Zorman P, Cimperman J, Lotric-Furlan S et al. (2002) Clinical and epidemiological findings for patients with erythema migrans: Comparison of cohorts from the years 1993 and 2000. Wien Klin Wochenschr 114: 493–497. PubMed: 12422589. 12422589

[11] Brouqui P, Bacellar F, Baranton G, Birtles RJ, Bjoërsdorff A et al. (2004) Guidelines for the diagnosis of tick-borne bacterial diseases in Europe. Clin Microbiol Infect 10: 1108–1132. doi:10.1111/j.1469-0691.2004.01019.x. PubMed: 15606643. 15606643

[12] Stanek G, Fingerle V, Hunfeld KP, Jaulhac B, Kaiser R et al. (2011) Lyme borreliosis: Clinical case definitions for diagnosis and management in Europe. Clin Microbiol Infect 17: 69–79. doi:10.1111/j.1469-0691.2010.03175.x. PubMed: 20132258. 20132258

[13] Cerar D, Cerar T, Ružić-Sabljić E, Wormser GP, Strle F (2010) Controlled prospective study on the development of subjective symptoms after treatment of early Lyme disease in Europe. Am J Med 123: 79–86. doi:10.1016/j.amjmed.2009.05.011. PubMed: 20102996. 20102996

[14] Ruzić-Sabljić E, Maraspin V, Lotric-Furlan S, Jurca T, Logar M et al. (2002) Characterization of *Borrelia* *burgdorferi* sensu lato strains isolated from human material in Slovenia. Wien Klin Wochenschr 114: 544–550. PubMed: 12422599. 12422599

[15] Harrell FE Jr, Lee KL, Pollock BG (1988) Regression models in clinical studies: determining relationships between predictors and response. J Natl Cancer Inst 80: 1199–1202. PubMed: 3047407. 10.1093/jnci/80.15.1198 3047407

[16] R Development Core Team (2009) R: A language and environment for statistical computing. R Foundation for Statistical Computing, Vienna, Austria ISBN 3-900051-07-0, URL. Available online at: http://www.R-project.org . Accessed March 22, 2011

[17] Aguero-Rosenfeld ME, Wang G, Schwartz I, Wormser GP (2005) Diagnosis of Lyme borreliosis. Clin Microbiol Rev 18: 484–509. doi:10.1128/CMR.18.3.484-509.2005. PubMed: 16020686. 16020686 PMC1195970

[18] Stanek G, Wormser GP, Gray J, Strle F (2012) Lyme borreliosis. Lancet 379: 461–473. doi:10.1016/S0140-6736(11)60103-7. PubMed: 21903253. 21903253

[19] Strle F, Nadelman RB, Cimperman J, Nowakowski J, Picken RN et al. (1999) Comparison of culture-confirmed erythema migrans caused by *Borrelia* *burgdorferi* sensu stricto in New York State and by *Borrelia* *afzelii* in Slovenia. Ann Intern Med 130: 32–36. doi:10.7326/0003-4819-130-1-199901050-00006. PubMed: 9890847. 9890847

[20] Logar M, Ruzić-Sabljić E, Maraspin V, Lotric-Furlan S, Cimperman J et al. (2004) Comparison of erythema migrans caused by *Borrelia* *afzelii* and *Borrelia* *garinii* . Infection 32: 15–19. doi:10.1007/s15010-004-3042-z. PubMed: 15007737. 15007737

[21] Strle F, Ružić-Sabljić E, Logar M, Maraspin V, Lotrič-Furlan S et al. (2011) Comparison of erythema migrans caused by *Borrelia* *burgdorferi* and *Borrelia* *garinii* . Vector Borne Zoonotic Dis 11: 1253–1258. doi:10.1089/vbz.2010.0230. PubMed: 21612533. 21612533

